# Purification and characterisation of the yeast plasma membrane ATP binding cassette transporter Pdr11p

**DOI:** 10.1371/journal.pone.0184236

**Published:** 2017-09-18

**Authors:** Katrine Rude Laub, Magdalena Marek, Lyubomir Dimitrov Stanchev, Sara Abad Herrera, Tamara Kanashova, Adèle Bourmaud, Gunnar Dittmar, Thomas Günther Pomorski

**Affiliations:** 1 Department of Plant and Environmental Sciences, University of Copenhagen, Frederiksberg C, Denmark; 2 Department of Molecular Biochemistry, Ruhr University Bochum, Bochum, Germany; 3 Mass spectrometry core unit, Max Delbrück Center for Molecular Medicine, Berlin, Germany; 4 Proteome and Genome research laboratory, Luxembourg Institute of Health, Strassen, Luxembourg; University of Cambridge, UNITED KINGDOM

## Abstract

The ATP binding cassette (ABC) transporters Pdr11p and its paralog Aus1p are expressed under anaerobic growth conditions at the plasma membrane of the yeast *Saccharomyces cerevisiae* and are required for sterol uptake. However, the precise mechanism by which these ABC transporters facilitate sterol movement is unknown. In this study, an overexpression and purification procedure was developed with the aim to characterise the Pdr11p transporter. Engineering of Pdr11p variants fused at the C terminus with green fluorescent protein (Pdr11p-GFP) and containing a FLAG tag at the N terminus facilitated expression analysis and one-step purification, respectively. The detergent-solubilised and purified protein displayed a stable ATPase activity with a broad pH optimum near 7.4. Mutagenesis of the conserved lysine to methionine (K788M) in the Walker A motif abolished ATP hydrolysis. Remarkably, and in contrast to Aus1p, ATPase activity of Pdr11p was insensitive to orthovanadate and not specifically stimulated by phosphatidylserine upon reconstitution into liposomes. Our results highlight distinct differences between Pdr11p and Aus1p and create an experimental basis for further biochemical studies of both ABC transporters to elucidate their function.

## Introduction

ATP binding cassette (ABC) transporters are members of a superfamily of proteins that mediate ATP-driven unidirectional transport of a variety of molecules across biological membranes. Proteins in this superfamily share a similar domain organization, with two membrane-embedded transmembrane domains (TMDs) and two cytoplasmic nucleotide binding domains (NBDs) with highly conserved amino acid motifs including the Walker A, the Walker B, and the signature C motifs. The four domains are often expressed as separate subunits in prokaryotic ABC proteins. In eukaryotes ABC proteins are organized either as full transporters comprising all four domains or as “half-transporters” with a single TMD and NBD that operate as homo- or heterodimers. Several ABC transporters utilise phospholipids among their substrates (reviewed in [1, 2]). Furthermore, some ABC transporters facilitate transport of sterols [1, 3]. Among these, there are two *Saccharomyces cerevisiae* ABC transporters, Aus1p and Pdr11p, which belong to the ABCG subfamily and are plasma-membrane-resident full-size transporters. Deletion of both AUS1 and PDR11 genes completely blocks sterol uptake under anaerobic conditions [4, 5] and in yeast mutants deficient in heme biosynthesis [6]. Under these conditions, *S. cerevisiae* is strictly dependent on the uptake of exogenously supplied sterols to proliferate as these lipids are essential for the cell, and their synthesis requires oxygen. The exact mechanism by which these ABC transporters facilitate sterol movement is unknown. Clarification of this issue is important as previous studies identified an orthologue of Aus1p in the pathogenic fungi *Candida glabrata* with an essential role in cholesterol uptake [7, 8]. *C. glabrata* can utilise exogenous cholesterol as a structural analogue and surrogate for ergosterol, thereby diminishing the effect of the ergosterol-specific antifungals.

At least three models, not mutually exclusive, have been proposed to explain how both proteins facilitate sterol uptake. One model is that Aus1p and Pdr11p control the initial insertion of sterol into the plasma membrane upon their passage across the cell wall [4, 6, 9]. In support of this, sterol uptake increases by coexpression of Aus1p and the cell wall protein Dan1p, which (like Aus1p and Pdr11p) is upregulated under anaerobic conditions [10]. Likewise, the presence of bovine serum albumin (BSA) promotes sterol uptake implying that BSA could act as a sterol donor [11]. A second model proposes that both proteins facilitate the removal of sterol from the plasma membrane to a cytosolic acceptor, such as soluble sterol-binding proteins, or closely apposed membranes of the endoplasmic reticulum [5]. Thirdly, Pdr11p and Aus1p have been proposed to flip sterols from the outer to the inner leaflet of the plasma membrane [5]), as spontaneous movement of sterols across the yeast plasma membrane is seemingly too slow for physiological processes to occur [12].

To investigate the function of these transporters, we previously established a purification procedure for Aus1p and reported its activity to be specifically stimulated by phosphatidylserine (PS) and blocked by the classical inhibitors of ABC proteins [13]. Here, we establish a procedure to purify Pdr11p and analysed its ATPase activity in terms of effects of inhibitors and requirements for lipids and sterols. Purified detergent-solubilised and reconstituted Pdr11p showed robust ATPase activity that was remarkably insensitive to orthovanadate and independent of lipid composition. This suggests that despite being part of the same physiological function, these two transporters may contain distinct and hitherto overlooked differences in their functionality.

## Materials and methods

### Materials

Synthetic dropout medium was from either Sigma-Aldrich (Taufkirchen, Germany) or Bio-Rad (Hercules, California). [*γ*-^32^P] ATP (3000 Ci/mmol) was from PerkinElmer (Waltham, Massachusetts). N-dodecyl-*β*-maltoside (DDM) was from GLYCON Biochemicals (Luckenwalde, Germany). Maltose neopentyl glycol-3 was a gift from Dr. Claus Løland, University of Copenhagen. All other detergents were obtained from Affymetrix (Maumee, Ohio, USA). PageRulerTM prestained or unstained protein ladder from Thermo Scientific (Waltham, Massachusetts) were used for size determination in SDS-PAGE gels. Lipids were purchased from Avanti Polar Lipids (Alabaster, AL). All other chemicals were from Sigma-Aldrich (Taufkirchen, Germany) unless otherwise indicated.

### Plasmid construction

The recombinant FLAG-tagged PDR11 expression plasmid was generated by amplification of the PDR11 ORF from genomic DNA by PCR using the primers 5’-GAGAGTCGACATGGATTACAAGGATGACGACGATAAAATCTCTCTTTCCAAATATTTTAATC-3’ and 5’-GAGAGCTAGCTTATACGCTTTGTTCGTTTGG-3’, and subsequent cloning of the amplified product into the Nhe1-Sal1 restriction enzyme sites of the pESC-URA expression plasmid (Agilent Technologies). The resulting expression vector was used as a template for mutagenesis of PDR11 by the QuickChange site-directed mutagenesis kit (Stratagene, La Jolla, CA) employing primers 5’-ATGGGTGAATCTGGTGCTGGT**ATG**ACAACTTTGTTGAATGTCTTG and 5’-CAAGACATTCAACAAAGTTGTCATACCAGCACCAGATTCACCCAT according to the manufacturer’s protocol to generate PDR11^K788M^. For making the FLAG-tagged PDR11-GFP expression plasmid, a PCR fragment containing the GFP tag was amplified from plasmid pYM27 (Euroscarf) using primers that contained homologous sequences for recombination with the Nhe1-digested vector pESC-URA-PDR11; *in vivo* recombination in yeast yielded plasmid pESC-URA-PDR11-GFP. All PCRs were carried out using Phusion High-Fidelity DNA Polymerase (New England Biolabs) according to the manufacturer’s instructions. All plasmid inserts were fully sequenced, including their junctions, to confirm the expected DNA sequence. The expression plasmid encoding FLAG-tagged Aus1p has been described previously [13].

### Yeast strains and growth conditions

Expression and functional complementation were carried out employing the sterol-uptake deficient *S. cerevisiae* mutant strain W303 *hem1*Δ::*LEU2 pdr11*Δ::*loxP aus1*Δ::*loxP-HIS5Sp-loxP*, with strain W303 *hem1*Δ*::LEU2* as wild-type [13]. Both strains were derivatives of W303-1*α* (*MAT*α* ade2-1 his3-11,15 leu2-3,112 trp1-1 ura3-1 can1-100*). In addition, the protease-deficient *S. cerevisiae* strain BJ1991 (*Mat*α* leu2 trp1 ura3-52 pep4-3 prb1-1122 gal2*) lacking Pep4p and Prb1p (yeast proteinases A and B) was used in expression studies. Yeast cells were transformed by the lithium acetate method [14] and grown at 30°C in selective synthetic dextrose (SD) or galactose (SG) medium lacking uracil. For the *hem1*Δ*aus1*Δ*pdr11*Δ strain SD medium was supplemented with 0.3% (w/v) adenine and 20 *μ*g/mL *δ*-aminolevulinic acid (ALA) while SG media was supplemented with 0.3% (w/v) adenine, 20 *μ*g/mL cholesterol and 0.05% (v/v) Tween 80. For solid media 2% (v/v) agar was added. For complementation tests, cells grown overnight in liquid SD medium were washed twice in sterile water and diluted to 0.2 OD_600_. Drops (3 *μ*L) of consecutive fivefold dilutions were spotted onto selective SG plates with ALA or cholesterol. The plates were incubated at 30°C for 4 days.

### Fluorescence microscopy and flow cytometry

Cells were examined under a Leica DMI4000 B inverted fluorescence microscope using a 63x water objective. GFP was visualised with an excitation filter BP 470/40, beam splitter 500, and BP 525/50 barrier filter. Flow cytometry of cells expressing GFP-tagged Pdr11p was performed as described previously [13].

### Membrane preparation and solubilisation

Yeast transformants from a liquid preculture were inoculated in 2 L of SD medium and grown at 30°C with shaking at 140 rev/min until an OD_600_ of 0.9-1.5 unless otherwise indicated. Cells were harvested, washed (1,000 g, 8 min, room temperature), and induced in 2 L SG medium for 6 h (unless otherwise stated). All subsequent steps were performed at 4°C. Cells were collected (1,000 g, 8 min) and lysed by vortexing 6 x 1 min with acid-washed glass beads (0.5 mm, BioSpec, Bartlesville, OK; 10 g beads per 5 g yeast) in lysis buffer (100 mM NaCl, 20 mM HEPES-NaOH, pH 7.4, 10 ml lysis buffer per 5 g yeast) containing 1 mM dithiothreitol (DTT), protease inhibitors (1 *μ*g/mL aprotinin, 1 *μ*g/mL leupeptin, 1 *μ*g/mL pepstatin, 5 *μ*g/mL antipain and 157 *μ*g/mL benzamidine, 0.25 mM phenylmethylsulfonyl fluoride) and PhosSTOP (Roche Applied Science, Penzberg, Germany). The lysate was cleared by centrifugation (1,000 g, 8 min) and the supernatant centrifuged again at 10,000 or 60,000 g (45 min) for collection of plasma membrane enriched fractions or total membranes in the pellet, respectively. The membrane pellets were homogenised with a douncer in 5 ml glycerol buffer (100 mM NaCl, 20 mM HEPES-NaOH, 20% (w/v) glycerol, pH 7.4) supplemented with protease inhibitors and adjusted to a protein concentration of 1 mg/mL (as measured by Bradford assay). Membranes were solubilised by detergent in the indicated concentrations for 1 h on an end-over-end rotator. Insoluble material was removed by centrifugation (88,000 g, 45 min) and the supernatant used for protein analysis.

### Protein purification and analysis

Plasma membrane fractions were isolated and solubilised in 0.6% (w/v) DDM (see above) and incubated with 14 *μ*l anti-FLAG (M2) affinity gel (prewashed twice in glycerol buffer) per 1 ml solubilised membrane followed by end-over-end rotation overnight at 16°C. The gel was washed three times with 25 ml glycerol buffer containing 0.05% (w/v) DDM. Finally the proteins were eluted by use of 400 *μ*g/mL FLAG peptide in glycerol buffer supplemented with 0.05% (w/v) DDM and protease inhibitors without phenylmethylsulfonyl fluoride, frozen in liquid nitrogen, and stored at -80°C. Aliquots of the eluate and cell lysates were analysed by Coomassie blue stained SDS-PAGE and Western blot using mouse monoclonal anti-FLAG M2 and anti-GFP (Roche) antibodies at 1:2000 dilution. Proteins were visualised with alkaline phosphatase-coupled secondary antibody using BCIP/NBT colour development substrate (Promega, Madison) according to manufacturer’s instructions. Concentration of the purified protein was determined by Coomassie Blue staining using bovine serum albumin as a standard via densitometry analysis performed on a Typhoon TRIO (GE Healthcare) equipped with a 633 nm laser (no emission filter) and analysed with ImageQuant TL (GE Healthcare).

### Mass spectrometric analysis

The protein preparation containing the recombinant Pdr11p was separated on an 8% SDS gel and stained with Coomassie Blue. Stained gel bands were cut into small pieces (about 1 x 1 mm) and converted to peptides as described by Shevchenko et al. [15]. Peptides were purified using C18 stage-tips according to Rappsilber et al. [16] and measured on a Q-Exactive HF mass spectrometer (Thermo Scientific) coupled to an Ultimate 3000 RSLCnano system (Thermo Scientific) in data-dependent acquisition mode, selecting the top 12 peaks for Higher Energy Collisional Dissociation (HCD) fragmentation. A 66 min gradient (solvent A: 0.1% formic acid; solvent B: HPLC grade acetonitrile in 0.1% formic acid) was applied for the samples using an Acclaim PepMap trap column (2 cm x 75 *μ*m i.d., C18, 3 *μ*m, 100 Å, Thermo Scientific) and an Acclaim PepMap RSLC analytical column (15 cm x 75 *μ*m i.d., C18, 2 *μ*m, 100 Å, Thermo Scientific). A volume of 3 μL sample was injected and the peptides eluted with 66 min gradients of 2 to 35% solvent B at flowrates of 0.3 μL/min. MS1 data acquisition was performed at a resolution of 60,000, using an injection time of 45 ms in the scan range from 375 to 1500 m/z and MS2 at a resolution of 15000 with an injection time of 45 ms. The normalized collision energy was set to 28 eV. The mass window for precursor ion selection was set to 1.2 m/z. The recorded spectra were analysed using the MaxQuant software package (Version 1.5.8.3) [17] by matching the data to the Uniprot yeast database (downloaded on 06.05.2012) with a false discovery rate of 1%.

### Protein reconstitution and vesicle analysis

Reconstitution in preformed liposomes was done as described in [18] using 52 mM octyl glycoside and a protein:lipid molar ratio of 1:50 or 1:100. Preformed liposomes were prepared from synthetic 1-palmitoyl-2-oleoyl (PO) phosphatidylcholine (PC), phosphatidylserine (PS), or phosphatidylglycerol (PG) and contained 0.1 mol% of the fluorescent marker lipid N-rhodamine-dioleoylphosphatidylethanolamine (Rho-PE). For floatation assays, 75 *μ*L proteoliposomes and 75 *μ*L 60% sucrose in lysis buffer were mixed. The sample was overlaid with 150 μL lysis buffer containing 25% sucrose, followed by 150 μL lysis buffer. Sucrose gradients were centrifuged (186,000 g, 1.5 h). Subsequently, three 150-μL fractions were collected (top, 0%; middle, 25%; bottom, 30%), and the pellet was resuspended in 150 μL of lysis buffer. Fluorescence of Rho-PE was detected on SDS/PAGE gels (Typhoon reader, excitation 532 nm, emission at 580 ± 30 nm), and proteins were visualised via silver staining. Lipid composition of the vesicles was confirmed by lipid extraction followed by thin-layer chromatography and lipid-phosphorus determination [19].

### ATPase assay

ATPase activity of Pdr11p was measured at 30°C for 30 min as described previously using 1 mM ATP, 5 mM MgCl_2_, 2 mM DTT, and 2 *μ*Ci of [*γ*-^32^P] ATP [13]. Release of inorganic phosphate was determined by indirect *β*-counting (1450 MicroBeta Trilux, Wallac). For determining the pH dependence of ATP hydrolysis, a range of different pH buffers were prepared: 50 mM MES for pH 5.3–6.6, 20 mM HEPES for pH 7–7.4, and 50 mM TRIS-HCl for pH 8–8.8. All were supplemented with 100 mM NaCl. When indicated MgCl_2_ was replaced by equal concentrations of MnCl_2_, CoCl_2_, or NiCl_2_. Cholesterol and ergosterol were added from an ethanolic stock to a final concentration of 6 *μ*g/mL in presence or absence of 30 *μ*g/mL bovine serum albumin.

### Data analysis

Background radiation was subtracted from the data. The ATPase activity versus orthovanadate data (including the 0 mM reference) have been fitted to the function *A* = *min* + (*max* − *min*)/(1+([*V*]/*IC*_50_)^−*H*^) where *A* is the ATPase activity, [*V*] is the orthovanadate concentration, *IC*_50_ is the half maximal inhibitory concentration, and *H* is the Hill coefficient. *IC*_50_, [*V*], *max*, and *min* values are all approximated with the open source program Gnuplot 4.6. Error bars show the standard deviations (S.D.). All relevant ATPase data are within the paper and its Supporting Information files (S1–S6 Tables).

## Results

### Overexpression of Pdr11p

**Fig 1:**
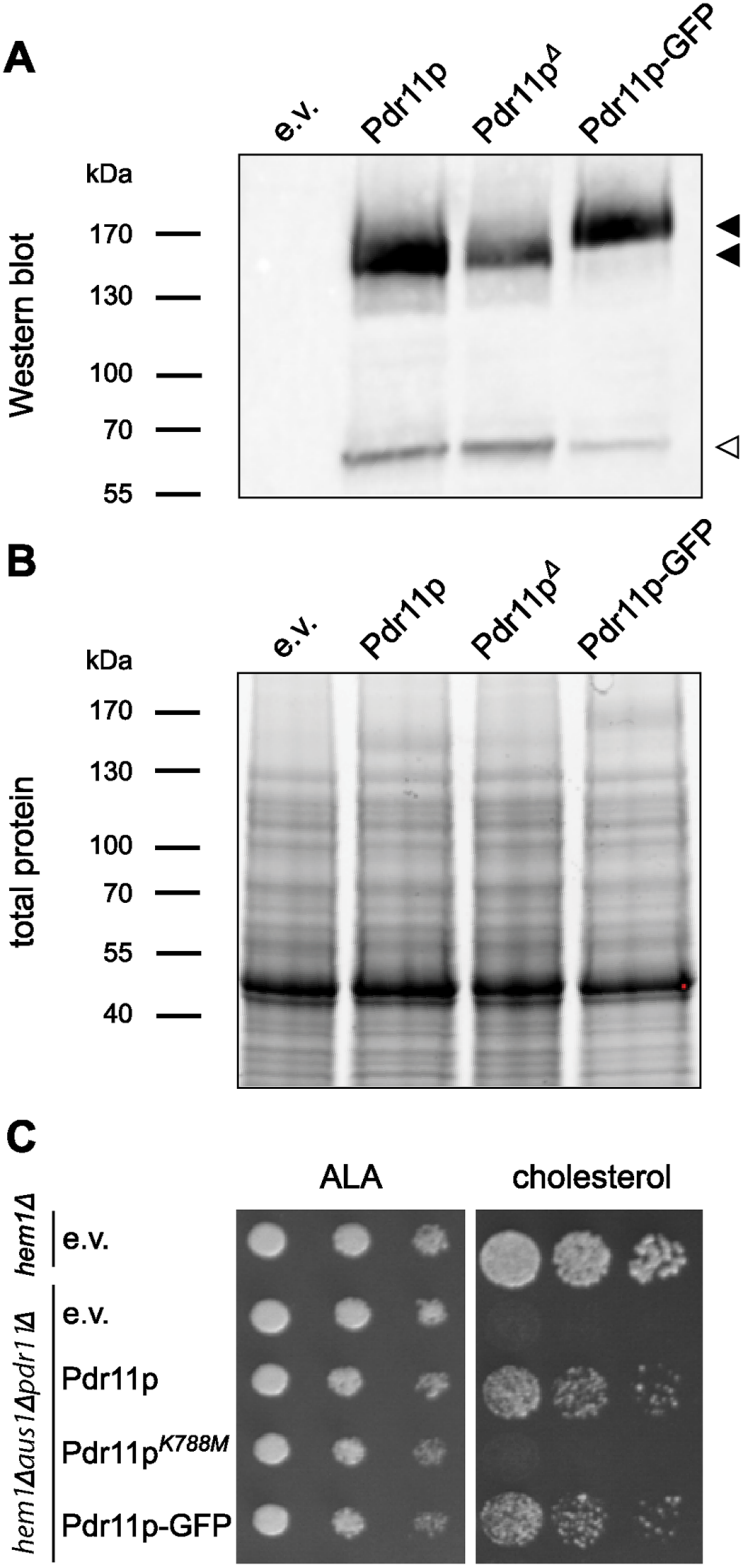
Overexpression and functionality of tagged Pdr11p variants. *S. cerevisiae* cells were transformed with empty vector (e.v.) or pESC-URA carrying *PDR11*, *PDR11-GFP*, or *PDR11^K788M^*. Sizes of the molecular mass standards are given to the left when relevant. A: Western blot illustrating the induced expression of FLAG-tagged variants of Pdr11p (filled arrowheads) and breakdown product (open arrowhead). Cells carrying the empty vector served as control. The blot was stained with anti-FLAG antibody. B: In-gel fluorescence detection using Criterion TGX stain-free gel. C: Serial dilutions of transformed heme-deficient *hem1*Δ and sterol uptake-deficient triple mutant *hem1*Δ*aus1*Δ*pdr11*Δ strains spotted onto standard synthetic galactose plates supplemented with *δ*-aminolevulinic acid (ALA) or cholesterol.

For expression analysis and purification of Pdr11p, three different 2-micron—based multi-copy plasmids were constructed to allow for galactose-inducible expression: a N-terminal FLAG-tagged Pdr11p (Pdr11p), a FLAG-tagged Pdr11p fused at the C terminus with green fluorescent protein (Pdr11p-GFP), and a catalytically inactive mutant of FLAG-tagged Pdr11p (Pdr11p^K788M^) carrying a lysine to methionine substitution in the Walker A region of NBD2 (Table 1) known to block the ATPase activity of ABC proteins [13, 20, 21]. To test the expression and functionality of the tagged proteins, we transformed the constructs into a sterol uptake-deficient mutant *hem1*Δ*aus1*Δ*pdr11*Δ. Upon galactose induction, immunoblotting of plasma membrane enriched extracts confirmed expression of the proteins (Fig 1A). All three variants of the protein were detected within the expected size range (FLAG-Pdr11p, 162 kDa; Pdr11p-GFP, 189 kDa). Furthermore, we routinely detected an additional band of approximately 60 kDa by the anti-FLAG antibody, indicating some proteolytic breakdown despite the presence of protease inhibitors. In-gel light-activated fluorescence demonstrated equal loading of the four lanes (Fig 1B). In addition, we employed growth complementation assay to verify the functionality of tagged Pdr11p and Pdr11p-GFP (Fig 1C). In line with their previously reported function in sterol transport [4, 5], both versions of the tagged proteins restored the growth defect of the triple mutant *hem1*Δ*aus1*Δ*pdr11*Δ on sterol-containing medium, while the mutated protein, Pdr11p^K788M^ was unable to sustain viability under these conditions. Collectively, these results confirm successful expression of functional Pdr11p and Pdr11p-GFP.

**Table 1:** Conserved ABC motifs of Aus1p and Pdr11p.

	Walker A	Signature	Walker B
**Aus1p-NBD1**	*GYPTSTLFKT*	VSGGE	*YLWDNST*
**Aus1p-NBD2**	GESGAGKTT	*LNPTQ*	LFLDEPT
**Pdr11p-NBD1**	*GNPTSALFKG*	VSGGE	*YLWDNST*
**Pdr11p-NBD2**	GESGAGKTT	*LSPTQ*	LFLDEPT

Amino acids are shown in single letter code. The sequence degeneracy of residues is highlighted in italic.

**Fig 2:**
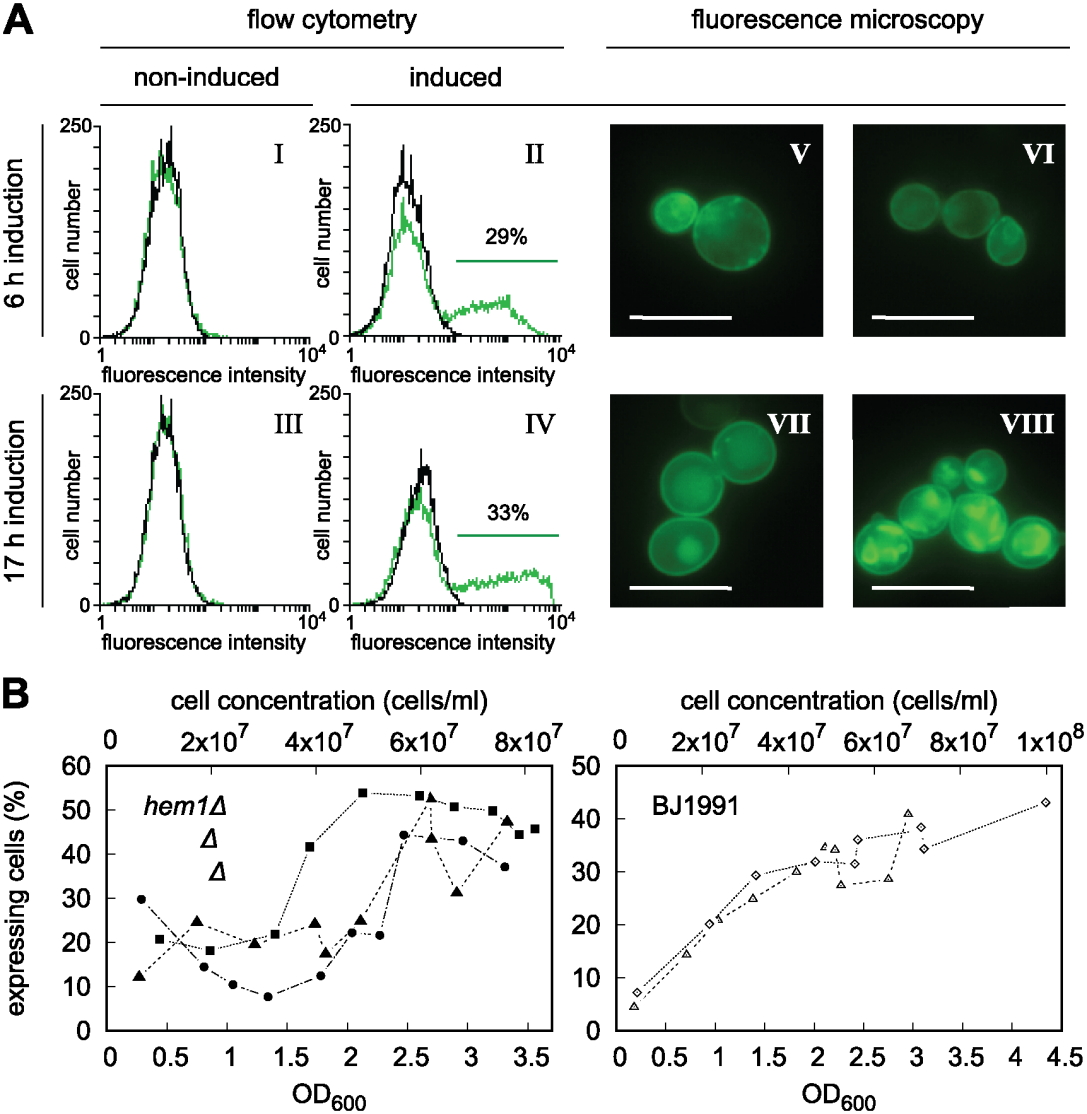
Expression analysis utilising GFP-tagged Pdr11p. A: Effect of induction time on Pdr11p-GFP expression in *hem1*Δ*aus1*Δ*pdr11*Δ. Panels I-IV, Representative flow cytometry based histograms of cells transformed with plasmids carrying *PDR11-GFP* (green) or empty vector (black) after 6 and 17 h in SD medium (non-induced) or SG medium (induced). Each data set consists of minimum 20,000 cells. Panels V-VIII, Representative fluorescence microscopy images of cells expressing Pdr11p-GFP after 6 and 17 h induction. The intensities of the fluorescent signals cannot be compared between the images. Bars equal 10 *μ*m. B: Percentage of cells expressing Pdr11p-GFP after galactose induction as a function of optical density at 600 nm (OD_600_) and cell concentration of the glucose pre-culture just prior to media change. All cultures in each repetition grew from a single colony. Lines are included solely to guide the eye. *hem1*Δ*aus1*Δ*pdr11*Δ strain, expression after 6 h induction in 3 cultures from the same selected high-expressing colony. BJ1991 strain, expression after 8 (open triangles) and 10 h (open diamonds) from the same selected colony.

To determine the optimal induction time, we followed the level of Pdr11p-GFP expression in *hem1*Δ*aus1*Δ*pdr11*Δ cells by flow cytometry and fluorescence microscopy (Fig 2). After 6 h of induction 36 ± 9% of the cells expressed the gene and the GFP-specific fluorescence was found at the plasma membrane and unidentified internal structures (Fig 2A, S7 Table). Increasing the induction time to 17 h resulted in a similar population (37 ± 5%) of expressing cells but caused an increased intracellular accumulation of the protein, indicating protein mistargeting and degradation. Analysis of cells grown from single colonies to different densities in repressing (SD) medium followed by culturing in inducing (SG) medium revealed a large variability in the number of expressing cells, i.e. induction of pre-cultures grown to OD_600_ <1 and >2.5 for 6 h resulted in 10–20% and 45% Pdr11p-GFP expressing cells, respectively (Fig 2B). A similar strong dependency in the number of expressing cells was also observed for the protease-deficient strain BJ1991 (Fig 2B, S8 Table). These results show a correlation between cell density/growth state in the repressing media and the level of Pdr11p-GFP expression in the inducing media. Thus, for all subsequent experiments, cells were therefore pre-cultured to OD_600_ >2.5 followed by induction for 6 h.

### Solubilisation and purification of Pdr11p

**Fig 3:**
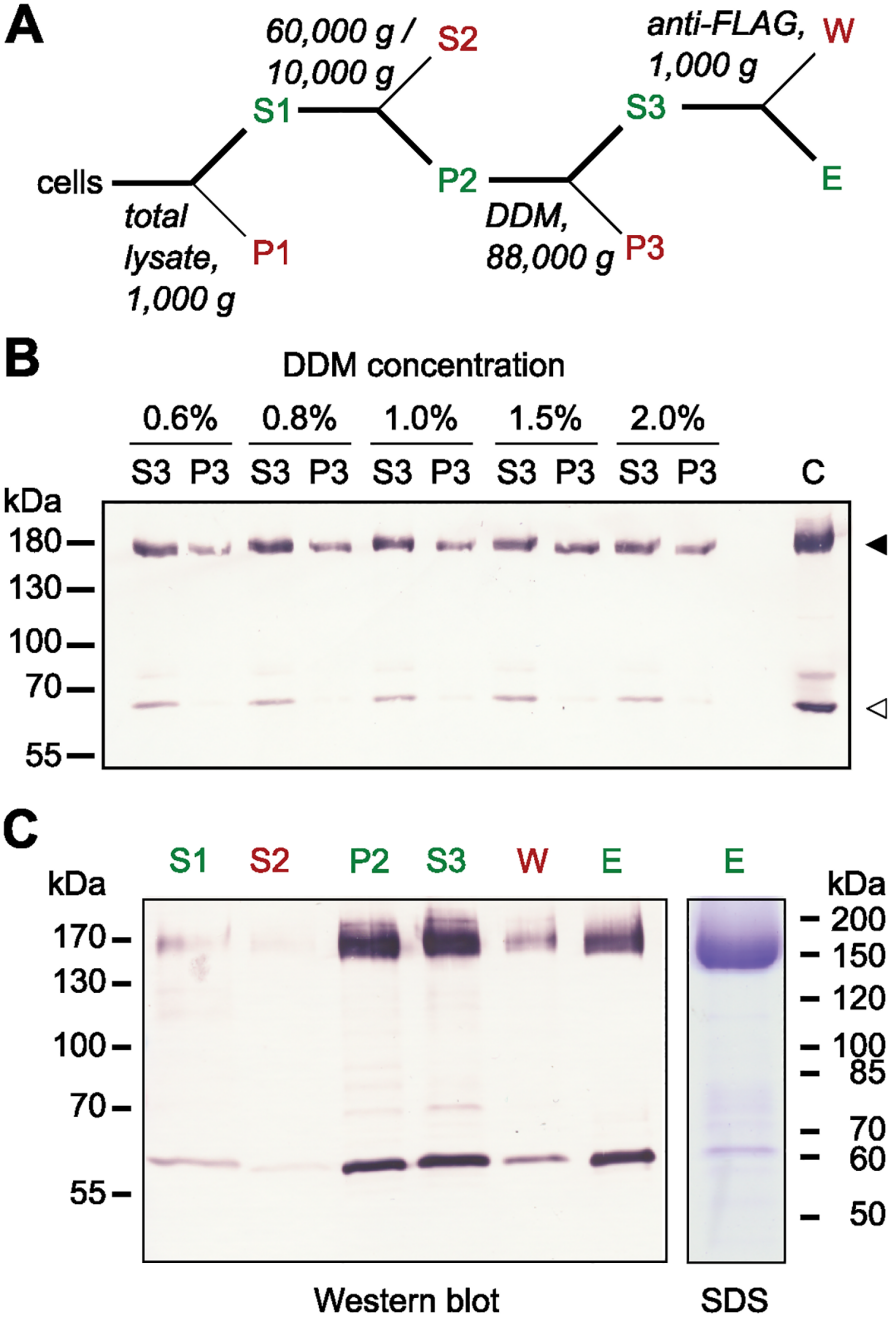
Solubilisation and purification of Pdr11p from *hem1*Δ*aus1*Δ*pdr11*Δ cells. A: Flowchart of the procedure. Supernatant S1 was centrifuged at 10,000 or 60,000 g (45 min) for collection of plasma membrane enriched fractions or total yeast membranes, respectively. B: Solubilisation of Pdr11p from total yeast membranes. Equal amounts of membranes (P2) were solubilised at the given DDM concentrations. After ultracentrifugation, the amounts of solubilised and non-solubilised Pdr11p were estimated in aliquots of supernatants (S3) and pellets (P3), respectively, via immunoblotting with anti-FLAG antibodies. Pdr11p, filled arrowheads; breakdown product, open arrowhead. C, original membrane fraction not been subjected to solubilisation (positive control). C: Purification of Pdr11p from plasma membrane enriched fractions. Representative Western blot analysis with anti-FLAG antibodies and Coomassie stained SDS-PAGE (SDS) of selected purification fractions. The loaded purification fractions are normalised with respect to volume. Sizes of the molecular mass standards are indicated on the sides.

For solubilisation, we prepared total membranes from Pdr11-GFP expressing cells (Fig 3A) and exposed them to a panel of nonionic and zwitterionic detergents. Among the tested detergents, we found DDM to be very effective in solubilising the protein even at a concentration of 0.6% (Fig 3B and 3C, lane P2 and S3) and consequently chose this mild non-ionic detergent for purification of Pdr11p from plasma membrane-enriched fractions by anti-FLAG affinity chromatography. Upon elution with a buffer containing FLAG peptide, the protein was highly enriched and readily detectable by Western blot analysis using anti-FLAG antibody and by Coomassie Blue staining (Fig 3C). Densitometric analysis of the Coomassie Blue-stained gels revealed at least a 95% degree of protein purity. We estimated the amount of purified protein to be up to 0.5 mg from 1 L culture, based on SDS-PAGE analysis along with standards of known amounts of bovine serum albumin. The preparation contains a second band migrating at 60 kDa. Mass-spectrometric analysis of the excised band at 160 kDa identified 47 peptides of Pdr11p (S9 Table).

### Functional characterisation of purified Pdr11p

Next, we analysed the ATPase activity of purified Pdr11p in detergent-containing buffer. To prevent possible interference from detergent, lipid, and reagents, we used an assay based on radiolabelled ATP. We estimated an ATPase activity of about 60 ± 24 nmol of ATP/min/mg of protein for the purified protein with Mg^2+^ as the catalysing metal ion. Pdr11p purified from the BJ1991 strain displayed a slightly reduced activity compared to protein purified from *hem1*Δ*aus1*Δ*pdr11*Δ cells (88 ± 19%, 2 independent measurements on the same purification).

**Fig 4:**
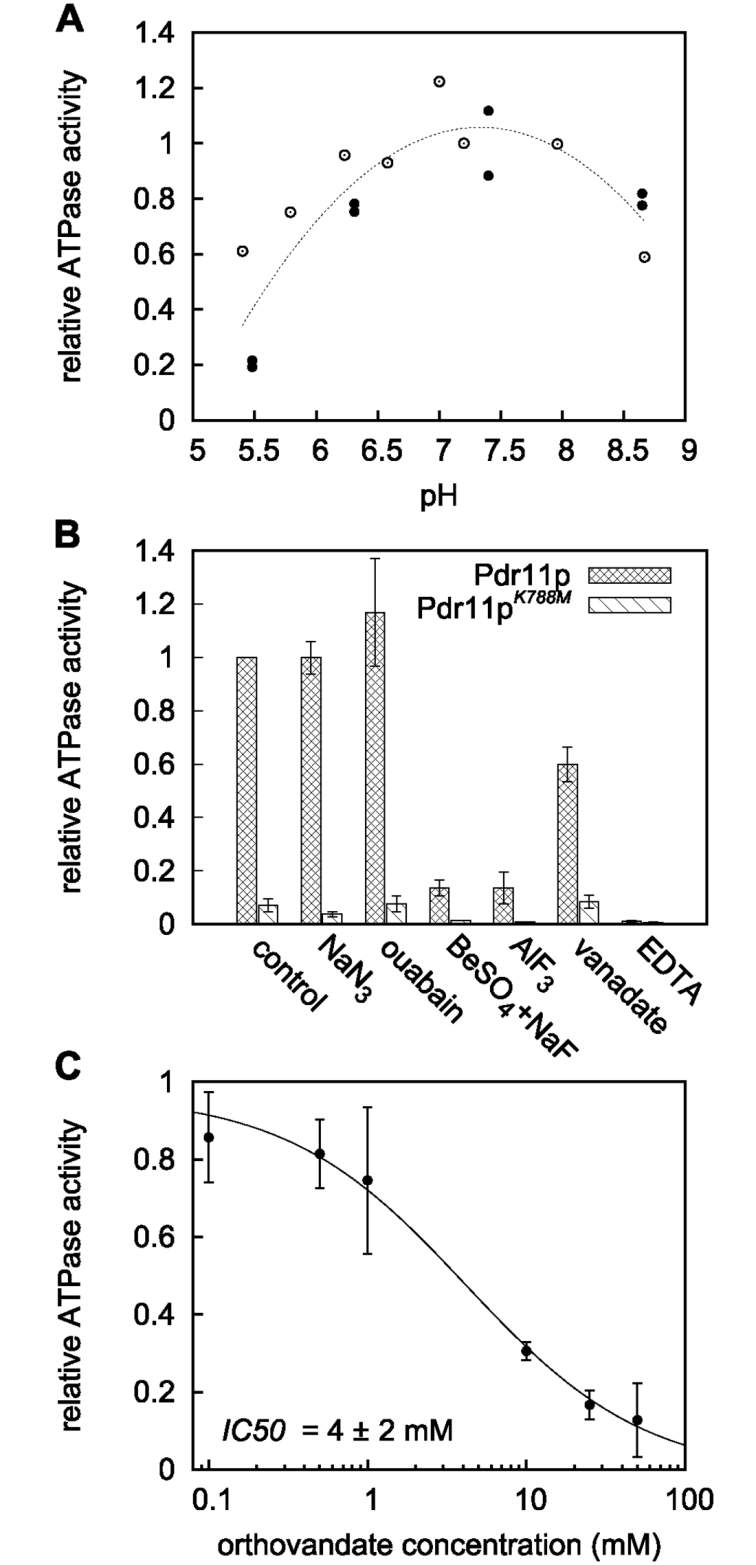
ATPase activity of solubilised Pdr11p. ATPase activity of the purified detergent-solubilised transporter was assayed as described under “Materials and Methods” using [*γ*-^32^P] ATP. A: ATPase activity as a function of pH. Open and filled circles are data from two independent experiments. Values are normalised with respect to the values at pH 7.2 (open circles) or pH 7.4 (closed circles). The dashed line is included to guide the eye. B: Effect of various inhibitors: NaN_3_, 5 mM; ouabain, 5 mM; BeSO_4_, 1 mM; NaF, 5 mM; AlF_3_, 1 mM; orthovanadate, 1 mM; EDTA, 1 mM. C: ATPase activity as a function of orthovanadate concentration. Fitting of data to a dose-response/activity curve (see Material and methods) gives *IC*_50_ = 4 ± 2 mM, and a Hill coefficient = 0.8 ± 0.2. Results in B and C are the mean ± S.D. from at least two independent experiments relative to the value obtained for the purified detergent-solubilised protein in the absence of inhibitors (control).

The purified transporter was active in a broad pH range (Fig 4A) with an optimum around 7.4 similar to other purified ABC transporters [22–24]. Depletion of Mg^2+^ by addition of EDTA abolished the activity demonstrating that ATP hydrolysis is dependent on the divalent cation. ATPase activity was unaffected in the presence of ouabain (an inhibitor of the Na^+^/K^+^-ATPase) or azide (an inhibitor of the F-ATPase) but was strongly inhibited by the classical inhibitors of ABC proteins beryllium fluoride and aluminium fluoride (Fig 4B). Based on this, we conclude that the observed ATPase activity is Pdr11p-dependent. Surprisingly, Pdr11p activity was barely sensitive to orthovanadate, with a calculated IC_50_ of 4 ± 2 mM (Fig 4C), which is about three orders of magnitude higher than other ABC transporters [23, 25–27]. The purified ATPase-inactive mutant Pdr11p^K788M^ contained comparable levels of contaminating proteins. A parallel analysis showed only very low background ATPase activity (7 ± 2% relative to wild type). We conclude that the observed ATPase activity is Pdr11p-dependent.

To ensure that the lack of vanadate inhibition was not due to slow binding, we preincubated the purified Pdr11p with 1 mM orthovanadate in presence of 1 mM ADP and analysed the ATPase activity upon addition of 1 mM ATP. The overall activity reduced to half (as expected in presence of equal amounts of ADP and ATP) while the low sensitivity towards vanadate inhibition remained (Table 2). Replacement of Mg^2+^ with other divalent cations can alter the activity and sensitivity to vanadate [28, 29]. We therefore repeated the ATPase assay in presence of MnCl_2_, NiCl_2_, and CoCl_2_. DTT was omitted from the experiments with the metal salts as it caused precipitation. Except Ni^2+^, all tested metal ions supported Pdr11p ATPase activity but with a maximal inhibition of little more than 50% for 1 mM vanadate neither Co^2+^ nor Mn^2+^ effectively altered the sensitivity to vanadate (Table 2).

**Table 2:** Effect of various compounds on ATPase activity of purified Pdr11p^a^.

Compound	Relative ATPase activity (%)	Inhibition by vanadate^b^ (%)
Control	100	27 ± 15
ADP	55 ± 30^c^	40 ± 4
Mn^2+^	150 ± 28^d^	29 ± 16
Co^2+^	41 ± 19^d^	54 ± 8
Ni^2+^	10 ± 19^d^	–^e^
Ergosterol	94 ± 5	ND
Cholesterol	105 ± 12	ND
Ergosterol/albumin	96 ± 1	ND
Cholesterol/albumin	104 ± 13	ND
Albumin	107 ± 5	ND

^a^ ATP hydrolysis of the purified detergent-solubilised transporter was assayed in the absence and presence of 1 mM orthovanadate. Indicated divalent cations replaced Mg^2+^. Data are based on at least duplicate determinations on one purification. ND, not determined.

^b^ Inhibition was expressed relative to assays containing the same divalent ion.

^c^ Relative to control without 1 mM ADP.

^d^ The divalent cation replaced Mg^2+^ in the assay buffer.

^e^ Activity too low to measure vanadate inhibition.

It is notable that addition of PhosSTOP to the ATPase assay with Mn^2+^ as a catalyst caused an increase in ATPase activity (150 ± 28%) that was not observed without PhosSTOP (82 ± 7%). The reasons are unclear at present.

Many ABC transporters have been shown to possess intrinsic ATPase activity that is stimulated in the presence of transported substrates [1, 24, 30]. As sterol uptake is mediated by Pdr11p [4] and facilitated by albumin [11], we tested the effect of ergosterol, cholesterol, and albumin on ATPase activity. Neither ergosterol nor cholesterol in absence and presence of albumin stimulated the ATPase activity of purified Pdr11p (Table 2).

### Analysis of vesicle-reconstituted Pdr11p

**Fig 5:**
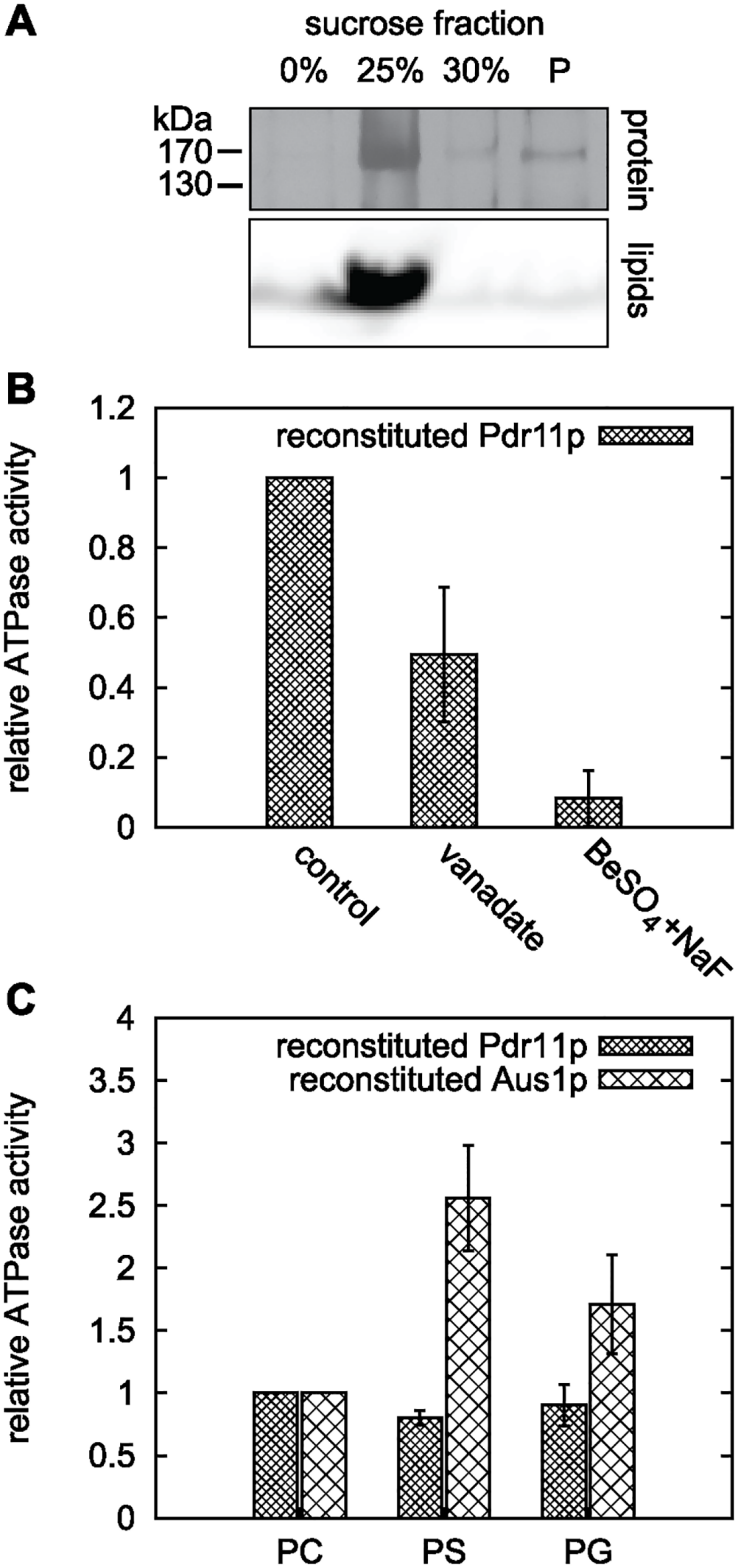
ATPase activity of liposome-reconstituted Pdr11p and Aus1p. Purified Pdr11p and Aus1p were reconstituted into different liposomes (containing Rho-PE as fluorescent lipid marker) and assayed for ATPase activity using [*γ*-^32^P] ATP. A: SDS PAGE analysis of a flotation assay of Pdr11p proteoliposomes in a sucrose gradient. Detection of lipids and protein in the same low density fraction validated successful reconstitution. Proteins are visualised by silver staining and lipids by fluorescence from Rho-PE. B: Relative ATPase activity of Pdr11p reconstituted in PS liposomes in presence of the indicated inhibitors: orthovanadate, 1 mM; BeSO_4_, 1 mM; NaF, 5 mM. Data is based on at least two reconstitutions from one purification batch. C: Lipid effect on ATPase activity of reconstituted Pdr11p and Aus1p. All activities are corrected for protein amount in the proteoliposomes. Data is based on two reconstitutions from one purification batch of each protein. PC, PC only; PS, PC/PS (1:1); PG, PC/PG (7:3).

To corroborate the effects of inhibitors and test the effect of the lipid environment, we reconstituted Pdr11p in unilamellar vesicles of different lipid compositions. In all cases we observed co-migration of the majority of Pdr11p and liposomes to the top of a sucrose gradient (Fig 5A), which confirms successful reconstitution. ATPase activity of liposome-reconstituted Pdr11p was assayed based on the release of inorganic phosphate from ATP as described for the purified protein. Similarly to the purified protein, lipid-reconstituted Pdr11p demonstrated robust ATPase activity that was efficiently inhibited by beryllium fluoride but not by orthovanadate (Fig 5B). ATPase activity was independent of lipid composition of the liposomes; Pdr11p reconstituted into PC, PC/PS, and PC/PG liposomes exhibited similar ATPase activity. Under the same conditions, reconstitution of Aus1 in PC/PS liposomes resulted in a stimulation of the ATPase activity, confirming previous results [13]. In addition, we observed a slight stimulation the ATPase activity of Aus1p upon reconstitution into PC/PG liposomes.

## Discussion

The study of membrane transporters at the molecular level is hampered by the complexity of native membranes in which they are embedded. Thus, an important step towards a detailed functional understanding of membrane proteins is their purification and reconstitution into defined, tuneable systems such as liposomes and nanodiscs. One important prerequisite for such analysis is access to adequate amounts of functional, purified protein. In this study, we describe a successful purification protocol and the biochemical characterisation of the yeast ABC transporter Pdr11p.

Expression of a FLAG tagged version of Pdr11p from a multicopy plasmid under the control of the strong inducible promoter, GAL10, allowed for effective purification to near homogeneity in yields high enough for subsequent biochemical studies. Further modification of Pdr11p—by addition of GFP fused at its C terminus—retained the functionality of the ABC transporter and facilitated the analysis of its expression and solubilisation. For expression, we tested two different host strains: the triple mutant strain W303 *hem1*Δ*aus1*Δ*pdr11*Δ and the protease deficient strain lacking yeast proteinases A (Pep4p) and B (Prb1p). In both strains, the number of expressing cells and intensity of the expression signal varied substantially for Pdr11p upon galactose induction. Several studies report similar results of heterogeneous expression within the cell culture for other proteins expressed from multicopy plasmids [11, 31–33]. A likely reason is fluctuating plasmid copy numbers within the cell population [34]. Interestingly, we observed a strong dependence of Pdr11p expression from the growth state of the cells in the repressing pre-culture medium; the highest expression was observed upon galactose induction of late logarithmic phase cells. In line with this, Mead et al. [34] observed less plasmid-free cells in cultures passing through the stationary phase. Microscopic analysis of the cells revealed that a short induction time was critical to prevent increased intracellular accumulation and degradation of Pdr11p-GFP.

Purification and reconstitution of Pdr11p into liposomes permitted us to study the biochemical properties of the transporter. Several biochemical features were similar to Aus1p [13]. First, purified Pdr11p exhibited a stable ATPase activity of about 60 ± 24 nmol of ATP/min/mg of protein even before reconstitution. For comparison, Aus1p has a specific activity of 56 nmol/min/mg under these conditions [13]. These activities correspond to ∼ 10 ATP molecules/min/protein and are within the range of values reported for a number of other purified eukaryotic ABC transporters, such as Ste6p, ABCR, transporter associated with antigen processing, and ABCA1 [35–38]. Second, mutagenesis of the conserved lysine to methionine (K788M) in the Walker A motif abolished ATP hydrolysis. Finally, neither ergosterol nor cholesterol stimulated the ATPase activity. In sharp contrast to Aus1p, whose activity is inhibited by low concentrations of orthovanadate and specifically stimulated by PS [13], Pdr11p ATPase activity was was barely sensitive to orthovanadate and not specifically stimulated by phosphatidylserine upon reconstitution into liposomes. These results highlight distinct differences between Pdr11p and Aus1p and create an experimental basis for further studies on both ABC transporters to elucidate their regulation by the lipid environment.

Similar to other fungal ABCG-family transporters Pdr11p and Aus1p have uniquely conserved asymmetric NBDs [39, 40]. The N-terminal NBD motif (NBD1) of both transporters contains a well-conserved ABC signature sequence, VSGGE, but possesses degenerated Walker A and Walker B motifs (Table 1). In contrast, the Walker A and Walker B motifs of the C-terminal NBD2 are conserved but the ABC signature motif is degenerated. The similarity of the ABC transporter motifs of Pdr11p and Aus1p indicates that an explanation for the different responses to orthovanadate should be found in other parts of the sequence (potentially around the Q-loop or H-loop) or by differences in the environment of the ATP binding sites. In line with this, a lack of orthovanadate inhibition has also been reported for other asymmetric ABC transporters such as ABCA1 [29] and ABCG5/G8 [41] and for symmetric ABC transporters such as ABCG1 [24], ruling out that this feature is connected to the non-consensus amino acids in one of the two binding sites. In some cases insensitive ABC transporters have recovered orthovanadate sensitivity by replacing Mg^2+^ with other divalent metal ions. This has been demonstrated for ABCA1 using Mn^2+^[29] and ABCC6 using Ni^2+^[28]. ABCG2 show orthovanadate-dependent adenine nucleotide trapping in the presence of Co^2+^ but not Mg^2+^[21]. None of the above ions restored orthovanadate sensitivity of Pdr11p. It remains to be established why some ABC transporters are only inhibited by beryllium and aluminium fluoride and not by orthovanadate.

In summary, we developed a relatively simple affinity-tag purification and reconstitution procedure yielding sufficient amounts of pure and active Pdr11p for future biochemical and biophysical studies that will potentially lead to elucidation of the mechanism(s) by which this transporter contributes to sterol uptake.

## Supporting information

S1 Table Data sets to Fig 4A. (DOCX)

S2 Table Data sets to Fig 4B. (DOCX)

S3 Table Data sets to Fig 4C. (DOCX)

S4 Table Data sets to Fig 5B. (DOCX)

S5 Table Data sets to Fig 5C. (DOCX)

S6 Table Data sets to Table 2. (DOCX)

S7 Table Figshare file information for Fig 2A. (DOCX)

S8 Table Figshare file information for Fig 2B. (DOCX)

S9 Table Protein identification by mass spectrometry. (DOCX)
