# Supplementary material for: Purification and characterisation of the yeast plasma membrane ATP binding cassette transporter Pdr11p
Source: PLoS One. 2017 Sep 18;12(9):e0184236. doi: 10.1371/journal.pone.0184236 (PMC5602531; doi:10.1371/journal.pone.0184236)
Supplement: S1 Table — (DOCX) [file pone.0184236.s001.docx]

**S1 Table. Data sets to Figure 4A.**

| **Experiment 1** | | |
| --- | --- | --- |
| pH | ATPase activity (cpm)^1^ | |
| 5.48 | 4.600 | 5.161 |
| 6.31 | 18.036 | 18.747 |
| 7.40 | 21.168 | 26.779 |
| 8.65 | 18.581 | 19.624 |
| **Experiment 2** | | |
| pH | ATPase activity (cpm)^1^ | |
| 5,40 | 4.598 | -- |
| 5,79 | 5.590 | -- |
| 6,23 | 7.044 | -- |
| 6,58 | 6.846 | -- |
| 7,00 | 8.918 | -- |
| 7,20 | 7.343 | -- |
| 7,96 | 7.324 | -- |
| 8,67 | 4.449 | -- |

^1^ For all measurements of ATPase activity background has been subtracted.
