# Supplementary material for: Purification and characterisation of the yeast plasma membrane ATP binding cassette transporter Pdr11p
Source: PLoS One. 2017 Sep 18;12(9):e0184236. doi: 10.1371/journal.pone.0184236 (PMC5602531; doi:10.1371/journal.pone.0184236)
Supplement: S2 Table — (DOCX) [file pone.0184236.s002.docx]

**S2 Table. Data sets to Figure 4B.**

| **Experiment** | | **1** | **2** | **3** |
| --- | --- | --- | --- | --- |
| Protein | Inhibitor | ATPase activity (cpm)^1^ | | |
| Pdr11 | none (control) | 26.820 | 39.044 | 21.168 |
|  | NaN3 | 27.930 | 37.271 |  |
|  | oubain | 27.494 | 51.199 |  |
|  | BeSO4+NaF | 3.091 | 6.089 |  |
|  | AlF3 | 2.517 | -- | 4.274 |
|  | vanadate | 14.794 | 25.124 |  |
|  | EDTA | 365 | 294 |  |
| Pdr11^K788M^ | none | 1.429 | 3.426 |  |
|  | NaN3 | 795 | 1.725 |  |
|  | oubain | 1.471 | 3.768 |  |
|  | BeSO4+NaF | 338 | 544 |  |
|  | AlF3 | 203 | -- |  |
|  | vanadate | 1.811 | 3.975 |  |
|  | EDTA | 208 | 92 |  |

^1^ For all measurements of ATPase activity background has been subtracted.
