# Supplementary material for: Purification and characterisation of the yeast plasma membrane ATP binding cassette transporter Pdr11p
Source: PLoS One. 2017 Sep 18;12(9):e0184236. doi: 10.1371/journal.pone.0184236 (PMC5602531; doi:10.1371/journal.pone.0184236)
Supplement: S3 Table — (DOCX) [file pone.0184236.s003.docx]

**Table S3. Data sets to Figure 4C.**

| **Experiment** | **1** | **2** |
| --- | --- | --- |
|  |  |  |
| Vanadate concentration (mM) | ATPase activity (cpm)^1^ | |
| 0 | 8.275 | 21.168 |
| 0 | 9.796 | 26.779 |
| 0,1 | 8.484 | 18.566 |
| 0,5 | 7.928 | 18.016 |
| 1 | 7.948 | 14.676 |
| 10 | 2.913 | 6.934 |
| 25 | 1.739 | 3.367 |
| 50 | 1.763 | 1.439 |

^1^ For all measurements of ATPase activity background has been subtracted.
