# Supplementary material for: Purification and characterisation of the yeast plasma membrane ATP binding cassette transporter Pdr11p
Source: PLoS One. 2017 Sep 18;12(9):e0184236. doi: 10.1371/journal.pone.0184236 (PMC5602531; doi:10.1371/journal.pone.0184236)
Supplement: S4 Table — (DOCX) [file pone.0184236.s004.docx]

**S4 Table. Data sets to Figure 5B.**

| **Experiment** | **1** | **2** | **3** | **4** |
| --- | --- | --- | --- | --- |
|  | ATPase activity (cpm)^1^ | | | |
| reconstituted Pdr11 (control) | 22.554 | 16.6952 | 37.947 | 11.507 |
| reconstituted Pdr11 + vanadate | 13.487 | 3.5192 | 23.879 | 6.256 |
| reconstituted Pdr11 + BeF | 3.153 | 4.404 |  |  |

^1^ For all measurements of ATPase activity background has been subtracted.
