# Supplementary material for: Purification and characterisation of the yeast plasma membrane ATP binding cassette transporter Pdr11p
Source: PLoS One. 2017 Sep 18;12(9):e0184236. doi: 10.1371/journal.pone.0184236 (PMC5602531; doi:10.1371/journal.pone.0184236)
Supplement: S5 Table — (DOCX) [file pone.0184236.s005.docx]

**S5 Table. Data sets to Figure 5C.**

| **Experiment** | **1** | | **2** | |
| --- | --- | --- | --- | --- |
|  | ATPase activity  (cpm)^1^ | Relative protein content^2^ | ATPase activity (cpm)^1^  (average of 2 measurements) | Relative protein content^2^ |
| Pdr11 PC | 38.108 | 1 | 22.751,0 | 1 |
| Pdr11 PS | 56.969 | 2,00 | 31.212,5 | 1,60 |
| Pdr11 PG | 56.353 | 2,01 | 33.090,5 | 1,36 |
| **Experiment** | **1** | | **2** | |
|  | ATPase activity  (cpm)^1^ | Relative protein content^2^ | ATPase activity  (cpm)^1^ | Relative protein content^2^ |
| Aus1 PC | 63.534 | 1 | 27.571 | 1 |
| Aus1 PS | 205.226 | 1,51 | 197.475 | 2,40 |
| Aus1 PG | 169.093 | 2,02 | 137.475 | 2,37 |

^1^ For all measurements of ATPase activity background has been subtracted.

^2^ Protein amount in the proteoliposomes was determined by SDS-PAGE analysis.
