# Supplementary material for: Purification and characterisation of the yeast plasma membrane ATP binding cassette transporter Pdr11p
Source: PLoS One. 2017 Sep 18;12(9):e0184236. doi: 10.1371/journal.pone.0184236 (PMC5602531; doi:10.1371/journal.pone.0184236)
Supplement: S6 Table — (DOCX) [file pone.0184236.s006.docx]

**S6 Table. Data sets to Table 2.**

| **Exp. No.** | **1** | **2** | **3** | **4** | **5** | **6** | **7** | **8** | **9** | **10** | **11** | **12** | **13** | **14** | **15** | **16** |
| --- | --- | --- | --- | --- | --- | --- | --- | --- | --- | --- | --- | --- | --- | --- | --- | --- |
|  | ATPase activity (cpm)^1^ | | | | | | | | | | | | | | | |
| Mg (control) | 101.041 | 9.945 | 11.135 | 43.654 | 75.588 | 7.612 | 16.494 | 10.822 | 12.734 | 20.676 | 38.481 | 18.148 | 6.098 | 284.654 | 77.562 | 113.069 |
| Mg + vanadate | 56.339 | 6.994 | 7.027 | 36.036 | 70.388 |  |  |  |  |  |  |  |  |  |  |  |
| ADP |  |  |  |  |  | 7.405 | 4.486 | 5.706 | 5.474 |  |  |  |  |  |  |  |
| ADP + vandate |  |  |  |  |  | 4.026 | 2.731 | 3.309 | 3.589 |  |  |  |  |  |  |  |
| Mn + phosstop | 211.041 |  |  |  |  | 10.573 |  |  |  | 37.488 |  |  |  |  |  |  |
| Mn - phosstop |  |  |  |  |  |  | 14.037 | 8.228 | 11.550 |  | 29.283 |  |  |  |  |  |
| Mn + vanadate | 35.423 |  |  |  |  |  | 11.959 | 4.002 | 6.102 |  |  |  |  |  |  |  |
| Co |  |  |  |  | 34.014 |  |  | 5.015 | 6.318 |  |  | 11.397 | 1.075 | 41.443 |  |  |
| Co + vanadate |  |  |  |  | 13.555 |  |  | 1.927 | 3.463 |  |  | 4.870 |  | 21.985 |  |  |
| Ni |  |  |  |  | 6.076 |  |  | 3.298 | 3.413 |  |  | 2.699 | 1.576 | 1.953 |  |  |
| ergosterol |  |  |  |  |  |  |  |  |  |  |  |  |  |  | 68.445 | 111.714 |
| cholesterol |  |  |  |  |  |  |  |  |  |  |  |  |  |  | 71.921 | 131.937 |
| ergosterol + albumin |  |  |  |  |  |  |  |  |  |  |  |  |  |  | 73.591 | 108.973 |
| cholesterol + albumin |  |  |  |  |  |  |  |  |  |  |  |  |  |  | 70.556 | 131.769 |
| albumin |  |  |  |  |  |  |  |  |  |  |  |  |  |  | 79.119 | 126.347 |

^1^For all measurements of ATPase activity background has been subtracted.
