# Supplementary material for: Purification and characterisation of the yeast plasma membrane ATP binding cassette transporter Pdr11p
Source: PLoS One. 2017 Sep 18;12(9):e0184236. doi: 10.1371/journal.pone.0184236 (PMC5602531; doi:10.1371/journal.pone.0184236)
Supplement: S7 Table — (DOCX) [file pone.0184236.s007.docx]

**S7 Table. Figshare file information for Fig 2A.** Flow cytometry files are available under Figshare DOI **10.6084/m9.figshare.5259457**. Explanation to the file names is given below.

|  |  |  |  |  |
| --- | --- | --- | --- | --- |
|  | **6 h induced** | | **17 h induced** | |
|  | **construct** | **file name** | **construct** | **file name** |
|  | empty vector | 6h_Pdr11GFP.001 | empty vector | 17h_Pdr11GFP.001 |
|  | Pdr11-GFP | 6h_Pdr11GFP.008 | Pdr11-GFP | 17h_Pdr11GFP.008 |
|  | Pdr11-GFP | 6h_Pdr11GFP.009 | Pdr11-GFP | 17h_Pdr11GFP.009 |
|  | Pdr11-GFP | 6h_Pdr11GFP.010 | Pdr11-GFP | 17h_Pdr11GFP.010 |
|  | Pdr11-GFP | 6h_Pdr11GFP.011 | Pdr11-GFP | 17h_Pdr11GFP.011 |
|  | Pdr11-GFP | 6h_Pdr11GFP.012 | Pdr11-GFP | 17h_Pdr11GFP.012 |
