# Supplementary material for: Purification and characterisation of the yeast plasma membrane ATP binding cassette transporter Pdr11p
Source: PLoS One. 2017 Sep 18;12(9):e0184236. doi: 10.1371/journal.pone.0184236 (PMC5602531; doi:10.1371/journal.pone.0184236)
Supplement: S8 Table — (DOCX) [file pone.0184236.s008.docx]

**S8 Table. Figshare file information for Fig 2B.** Flow cytometry files are available under Figshare DOI **10.6084/m9.figshare.5259454**. Explanation to the file names is given below.

|  |  | | | | | |
| --- | --- | --- | --- | --- | --- | --- |
|  | ***hem1Δaus1Δpdr11Δ *strain** | |  | |  | |
|  | **1st set** | | **2nd set** | | **3rd set** | |
|  | **OD** | **file name** | **OD** | **file name** | **OD** | **file name** |
|  | 0,77 | OD0.77 control 28-4-16**^1^** | 0,74 | OD0.74 control 250516**^1^** | 0,82 | OD0.82 control 80616**^1^** |
|  | 0,29 | OD0.29 28-04-16.001 | 0,27 | OD0.27 250516.001 | 0,44 | OD0.44 080616.001 |
|  | 0,81 | OD0.81 28-04-16 | 0,75 | OD0.75 250516 | 0,86 | OD0.86 080616 |
|  | 1,05 | OD1.05 28-04-16 | 1,23 | OD1.23 250516 | 1,4 | OD1.4 080616 |
|  | 1,34 | OD1.34 28-04-16 | 1,73 | OD1.73 250516 | 1,69 | OD1.69 080616 |
|  | 1,78 | OD1.78 28-04-16 | 1,82 | OD1.82 250516 | 2,6 | OD2.6 080616 |
|  | 2,27 | OD2,27 28-04-16 | 2,7 | OD2.7 250516 | 2,13 | OD2.13 080616 |
|  | 2,04 | OD2.04 28-04-16 | 2,11 | OD2.11 250516 | 2,89 | OD2.89 080616 |
|  | 2,47 | OD2.47 28-04-16 | 2,69 | OD2.69 250516 | 3,21 | OD3.21 080616 |
|  | 2,96 | OD2.96 28-4-16 | 2,91 | OD2.91 250516 | 3,43 | OD3.43 080616 |
|  | 3,31 | OD3.31 28-4-16 | 3,33 | OD3.33 250516 | 3,56 | OD3.56 080616 |

**^1^** Cells that were not induced in SG medium but cultured in SD medium.

|  | **BJ1991 strain** | |  | |  | |
| --- | --- | --- | --- | --- | --- | --- |
|  | **6h** | | **8h** | | **10h** | |
|  | **OD** | **file name** | **OD** | **file name** | **OD** | **file name** |
|  | 0,32 | 6hcOD0.32_6h control**^1^** |  |  |  |  |
|  | 0,18 | 6hOD0.18_6h.001 | 0,18 | 8hOD0.18_8h.001 | 0,21 | 10hOD0.21_10h.001 |
|  | 0,52 | 6hOD0.52_6h | 0,71 | 8hOD0.71_8h | 0,94 | 10hOD0.94_10h |
|  | 0,6 | 6hOD0.6_6h | 1,03 | 8hOD1.03_8h | 1,41 | 10hOD1.41 |
|  | 0,96 | 6hOD0.96_6h | 1,38 | 8hOD1.38_8h | 2,01 | 10hOD2.01_10h |
|  | 1,11 | 6hOD1.11_6h | 1,82 | 8hOD1.82_8h | 2,41 | 10hOD2.41_10h |
|  | 1,41 | 6hOD1.41_6h | 2,11 | 8hOD2.11_8h | 2,44 | 10hOD2.44_10h |
|  | 1,64 | 6hOD1.64_6h | 2,21 | 8hOD2.21_8h | 3,05 | 10hOD3.05_10h |
|  | 2,08 | 6hOD2.08_6h | 2,27 | 8hOD2.27_8h | 3,08 | 10hOD3.08_10h |
|  | 2,29 | 6hOD2.29_6h | 2,75 | 8hOD2.75_8h | 3,11 | 10hOD3.11_10h |
|  | 2,31 | 6hOD2.31_6h | 2,95 | 8hOD2.95_8h | 4,34 | 10hOD4.34_10h |

**^1^** Cells that were not induced in SG medium but cultured in SD medium.
