# Supplementary material for: Purification and characterisation of the yeast plasma membrane ATP binding cassette transporter Pdr11p
Source: PLoS One. 2017 Sep 18;12(9):e0184236. doi: 10.1371/journal.pone.0184236 (PMC5602531; doi:10.1371/journal.pone.0184236)
Supplement: S9 Table — (DOCX) [file pone.0184236.s009.docx]

**S9 Table. Protein identification by mass spectrometry.**

|  |  | **66 kDa band** | | **160 kDa band** | |
| --- | --- | --- | --- | --- | --- |
| **Protein^1^** |  | **Sequence coverage**  **(%)** | **# peptides** | **Sequence coverage (%)** | **# peptides** |
| P40550 | ATP-dependent permease PDR11 | 5,7 | 7 | 36,5 | 47 |
| P05030 | Plasma membrane ATPase 1 | 0 | 0 | 1,1 | 1 |
| P53064 | RNA polymerase-associated protein RTF1 | 14,3 | 7 | 1,8 | 1 |
| P10592 | Heat shock protein SSA2 | 27,4 | 14 | 0 | 0 |
| P27614 | Carboxypeptidase S | 5,9 | 3 | 0 | 0 |
| P40988 | Low-affinity Fe(2+) transport protein | 3,6 | 2 | 0 | 0 |
| P60010 | Actin | 6,9 | 2 | 0 | 0 |
| Q99220 | Protein OS-9 homolog | 1,7 | 1 | 0 | 0 |

^1^ Protein preparation containing the recombinant Pdr11p was separated on an 8% SDS gel and stained with Coomassie Blue. Stained gel bands were cut into small pieces (about 1 x 1 mm), converted to peptides and analysed on a Q-Exactive mass spectrometer as described under Materials and methods.
